# Hospital length of stay for COVID-19 patients: Data-driven methods for forward planning

**DOI:** 10.1186/s12879-021-06371-6

**Published:** 2021-07-22

**Authors:** Bindu Vekaria, Christopher Overton, Arkadiusz Wiśniowski, Shazaad Ahmad, Andrea Aparicio-Castro, Jacob Curran-Sebastian, Jane Eddleston, Neil A Hanley, Thomas House, Jihye Kim, Wendy Olsen, Maria Pampaka, Lorenzo Pellis, Diego Perez Ruiz, John Schofield, Nick Shryane, Mark J. Elliot

**Affiliations:** 1 Department of Social Statistics, School of Social Sciences, University of Manchester, Oxford Road, Manchester, M13 9PL UK; 2 Department of Mathematics, University of Manchester, Oxford Road, Manchester, M13 9PL UK; 3 Division of Informatics, Imaging and Data Science, Faculty of Biology, Medicine and Health, University of Manchester, Manchester Academic Health Science Centre, Oxford Road, Manchester, M13 9PL UK; 4 IBM Research, Hartree Centre, Daresbury, UK; 5 Division of Diabetes, Endocrinology & Gastroenterology, School of Medical Sciences, Faculty of Biology, Medicine & Health, University of Manchester, Oxford Road, Manchester, M13 9PT UK; 6 Clinical Data Science Unit, Manchester University NHS Foundation Trust, Oxford Road, Manchester, M13 9WU UK; 7 Department of Mathematics, University of Liverpool, Peach Street, Liverpool, L69 7ZL UK; 8 Department of Virology, Manchester Medical Microbiology Partnership, Manchester Foundation Trust, Manchester Academic Health Sciences Centre, Oxford Road, Manchester, M13 9WU UK

**Keywords:** COVID-19, Length of stay, Survival Analysis, England

## Abstract

**Background:**

Predicting hospital length of stay (LoS) for patients with COVID-19 infection is essential to ensure that adequate bed capacity can be provided without unnecessarily restricting care for patients with other conditions. Here, we demonstrate the utility of three complementary methods for predicting LoS using UK national- and hospital-level data.

**Method:**

On a national scale, relevant patients were identified from the COVID-19 Hospitalisation in England Surveillance System (CHESS) reports. An Accelerated Failure Time (AFT) survival model and a truncation corrected method (TC), both with underlying Weibull distributions, were fitted to the data to estimate LoS from hospital admission date to an outcome (death or discharge) and from hospital admission date to Intensive Care Unit (ICU) admission date. In a second approach we fit a multi-state (MS) survival model to data directly from the Manchester University NHS Foundation Trust (MFT). We develop a planning tool that uses LoS estimates from these models to predict bed occupancy.

**Results:**

All methods produced similar overall estimates of LoS for overall hospital stay, given a patient is not admitted to ICU (8.4, 9.1 and 8.0 days for AFT, TC and MS, respectively). Estimates differ more significantly between the local and national level when considering ICU. National estimates for ICU LoS from AFT and TC were 12.4 and 13.4 days, whereas in local data the MS method produced estimates of 18.9 days.

**Conclusions:**

Given the complexity and partiality of different data sources and the rapidly evolving nature of the COVID-19 pandemic, it is most appropriate to use multiple analysis methods on multiple datasets. The AFT method accounts for censored cases, but does not allow for simultaneous consideration of different outcomes. The TC method does not include censored cases, instead correcting for truncation in the data, but does consider these different outcomes. The MS method can model complex pathways to different outcomes whilst accounting for censoring, but cannot handle non-random case missingness. Overall, we conclude that data-driven modelling approaches of LoS using these methods is useful in epidemic planning and management, and should be considered for widespread adoption throughout healthcare systems internationally where similar data resources exist.

**Supplementary Information:**

The online version contains supplementary material available at (10.1186/s12879-021-06371-6).

## Background

Since its emergence in December 2019 and classification in January 2020, SARS-CoV-2, the coronavirus that causes COVID-19, has spread rapidly, with 270 thousand confirmed infections in the UK by the end of May 2020 [1]. The exponential growth in the early days of each nation’s outbreak has led to a doubling time of around three days [2]. Coupled with potentially high estimates of *R*_0_ (the average number of new infections generated by an infected individual, in the absence of control measures and population acquired immunity) [3–5], this has continued to have substantial impacts on healthcare systems across the world. Large growth rates and a delay between new infections and their detection can lead to unexpected surges in bed demand. In order to restrict the spread of the pathogen, many countries have implemented mass quarantine (also known as lockdown) strategies, including England where the mass quarantine began on 23 March 2020 [6]. However, the effects of such interventions are not seen for at least a week [7], emphasising the need for careful, evidence-based, planning; particularly as the easing of mass quarantine measures is considered. In this context, the use of clinical care data to predict the demand for hospital and Intensive Care Unit (ICU) beds by patients presenting with COVID-19 is invaluable in optimising the effectiveness of planning by hospitals and, therefore, patient outcomes.

Understanding the impact of COVID-19 on hospital capacity breaks down into two core measurement tasks: first, to predict incidence (and thereby hospital admissions rates); and second, to estimate total length of stay (LoS) accurately allowing for variation in severity of disease and healthcare needs. The combination of these two measures can then be used to predict bed demand. This challenging task requires a careful modelling approach, particularly when high-quality data is limited within often fragmented healthcare systems. National datasets are crucial in understanding demand in hospitals across the country, but are flawed by amounts of record-level (or whole case) missingness that can bias the estimates. Routinely collected data generated by individual hospitals are, by definition, smaller and non-general but tend to be less prone to missingness and these can complement national data by providing insights for planning on a local level.

Estimating LoS has not been the primary focus of previous modelling; and studies that calculate LoS tend to use ad-hoc approaches [8]. There is currently a lack of statistically principled modelling that accounts for both delays in patient outcomes and complex hospitalisation pathways. This problem is particularly important during the COVID-19 pandemic, since some groups of patients spend extended periods in hospital, and, for the most severe cases, in critical care. Furthermore, estimates of LoS that use deterministic models or observations drawn directly from data fail to take missingness into account [9–11]. Accurately calculating LoS therefore requires mathematical and statistical techniques that specifically address these issues.

In this paper, we present three methods for estimating LoS for patients with COVID-19 infection using both a nationally collected dataset and local data from a large inner city hospital Trust in the UK. The truncation corrected (TC) method corrects for the fact that observations are truncated at the day of reporting; accelerated failure time models (AFT) explicitly account for all observed LoS including those censored by not having seen the outcome; and the multi-state (MS) approach analyses LoS and takes into account dependence between outcomes such as discharge or death. Finally, we include measures of uncertainty in each of our model results, which should be incorporated into hospital planning strategies. With this principled approach, past data can be appropriately used to better prepare for the next phase of the COVID-19 pandemic.

The results presented in this article use data that were available as of 26 May 2020. At this stage of the pandemic, many patients were still in hospitals, leading to right-censoring in their lengths of stay. To evaluate the performance of the methods at correcting for this right-censoring, we compare the estimated distribution to the full LoS distributions, using data available as of 21 January 2021. We do not re-analyse the LoS for the second and third waves, since this manuscript focuses on comparing methods for estimating LoS whilst correcting for right-censoring. However, the methods are readily applicable to these more recent data.

## Methods

### Data

#### Outcome variables

We define two outcome events: death or discharge. All patients admitted to hospital will eventually experience one of these two outcomes. Then, we model LoS from hospital admission to either death or discharge. For the analysis shown in the “Results” section, we focus on LoS until any outcome, to facilitate comparison of the three methods. We account for whether the patient was in ICU or not and also estimate the LoS from hospital admission to ICU and LoS on ICU. In Appendix F, we further examine different outcomes using the TC and MS methods.

#### CHESS

The COVID-19 Hospitalisation in England Surveillance System (CHESS)^1^ collects reports from all NHS acute care hospital trusts to provide daily patient-level and aggregate data on COVID-19 hospitalisations. In the patient-level data, patients are followed through their hospitalisation pathway; the dates of various events are recorded, such as date of admission to hospital, date of admission to ICU and final outcome date.

#### CHESS predictors

We used four variables as predictors. First, *sex*, for which we removed patients with unknown values. Second, *age*, which we grouped into four categories (<50,50−64,65−74,75+), and removed negative values and patients with a recorded age equal to zero (which did not seem genuine, based on the number of such cases and other factors such as comorbidities). Third, *week of admission to hospital*, which, in the TC model, we categorised in two groups: weeks 12 to 14 (i.e. from 16 March to 5 April 2020), and weeks 15 to 20 (from 6 April to 17 May 2020). In the AFT model, we used single week as a fixed effect predictor but present results for the two groups of admissions. Fourth, we used a binary indicator on whether a patient was admitted to ICU or not, and omitted the patients for whom this information was unknown. The resulting analytical sample is *n*=6208. Details of the data processing procedure, and inclusion/exclusion criteria, are presented in Appendix C.

Whilst we can identify predictors such as sex, age, and week-of-admission from these data, we cannot identify other potential predictors such as which variant contributed to the infection or treatment strategies. This would be of interest with the emergence of new variants of concern. Instead, the effect of new variants has to be approximated using week-of-admission, but this may be confounded with other factors, such as treatment changes and hospital burden.

#### Routinely collected hospital data (MFT)

Routine data on the hospitalisation of patients were provided by Manchester University NHS Foundation Trust (MFT). MFT is the largest NHS Trust in England, comprising nine hospitals and accounting for approximately 2.5% of the National Health Service. For COVID-19 admission, there were three geographically distinct acute hospitals across South and Central Manchester: Manchester Royal Infirmary; Wythenshawe Hospital; and Trafford General Hospital. MFT serves the population of Greater Manchester, a large, ethnically diverse conurbation of approximately 2.8 million people. The data follow all patients through their clinical pathway for the duration of a single hospitalisation, and provide timings and lengths of stay in all critical care episodes. Patient data are complete unless patients are still in hospital, in which case they are censored.

#### MFT data preparation

Data were drawn from the Patient Administration System (PAS) and WardWatcher to join information on a patient’s hospitalisation pathway and critical care episodes. Patients were selected from the MFT database if a swab was taken either on the day of their hospitalisation, or within two days of their hospital admission, and tested positive for COVID-19. This was to discount any hospital-acquired cases since COVID-19 positive cases who required hospitalisation due to non-COVID related health conditions may bias LoS estimates. We also excluded patients admitted for elective procedures requiring treatment for chronic illnesses such as dialysis. As a result of having multiple admissions close together, it was difficult to determine whether these cases were hospital-acquired or genuine COVID-19 admissions. The resulting sample included *n*=786 patients. The models based on the MFT data did not use information on predictors due to the smaller sample size, although from a methodological point of view these could be easily added to the models. Details of the data generating process are presented in Appendix A.

### Data quality issues in length of stay data

There are several types of data quality issues that tend to be present in length of stay data and all are present in one or both of the two datasets. Some of these are a consequence of the reporting and data collection methods. Others are inherent to the nature of outbreaks, and will be present regardless of the data collection. Here, we present some key issues that need to be adjusted for, and discuss the implications of ignoring them. Accounting for these biases for COVID-19 can enable robust estimates that provide timely insight for policy and planning.

#### Missing cases

One issue with the CHESS dataset is missing cases. For example, the number of deaths recorded in CHESS is considerably less than the official figures. These also suffer from reporting lag issues but some indication about the level of missingness in CHESS can be obtained by comparing to the COVID-19 patient notification system (CPNS), which records all deaths attributed to COVID-19 in England. On 26 May, there were 23504 deaths in hospital as attributable to COVID-19 in the CPNS data. This compares to an equivalent figure of 4071 in the raw CHESS data for the same day. This is indicative of case level missingness within CHESS of over 80%. We discuss this issue in more detail in “Discussion” section.

#### Missing values on important variables

Many rows in the data are incomplete. This is particularly problematic for data pertaining to outcome events: for example in some cases it is unclear whether a patient has not been discharged yet, or whether they have but the data have not been recorded. The amount of, and patterns of, missing patient information in the CHESS data is associated with the trust that reports the cases, with varying levels of missingness across different trusts (see Appendix B).

#### Censoring

In time-to-event studies, we observe a collection of individuals who are infected or have been exposed to infectious material. If these individuals could be followed indefinitely, the outcomes of all individuals would be observed. Therefore, these data can be used to determine the length of stay in the various compartments (states) of the disease progression pathway, as well as the probabilities of transitions into other states. However, during an outbreak we only observe individuals up until the most recent reporting date. This leads to right-censoring (e.g. [12]), when we only know the lower bound of duration until the next event in the pathway, and cannot accurately determine the length of time until their next transition nor to which state this will be. Thus, censoring may lead to the underestimation of the LoS.

#### Truncation bias

To remove the uncertainty around censored cases, we can instead condition our sample to only look at cases for whom the outcome has been observed. However, such a sample includes only cases with outcomes that occurred before the most recent reporting date, causing the sample to be truncated by the reporting date. This truncation leads to an over-expression of short LoS, since the recently infected individuals are only included if their LoS is short. Failing to account for this bias will underestimate the LoS of interest^2^.

Truncation is exacerbated by exponential growth in the early stages of an outbreak, since a higher proportion of cases will have been infected recently. By the final phase of an outbreak, truncation has a smaller effect since the majority of cases occurred sufficiently long ago to be unaffected by the truncation date. However, it will always be present as long as the epidemic is ongoing. Even in these late stages, whilst it may have a negligible impact across the whole outbreak, its effect might be of concern in certain scenarios, such as when using time as a predictor variable. In such a case, for events early in the epidemic, truncation will have very little effect, but for more recent events many cases may still be truncated. Such biases are often considered in the HIV literature [13, 14], due to the long infectious periods involved, but are often ignored for acute outbreaks. As alluded to in [15], this is potentially due to high quality data being available only after an explosive outbreak has finished, by which point these biases have little or no effect. However, when attempting to control ongoing epidemics, we require estimates of LoS distributions that are robust in the face of censoring and truncation.

### Survival analysis

Survival analysis describes a collection of statistical procedures for which the outcome of interest is time until an event, often as a function of predictor variables [16–18]. A central assumption of most survival analytic methods is that the time to event will have been censored for some observations, as discussed in “Data quality issues in length of stay data” section.

Survival analysis may assume an underlying distribution for LoS in each state. Generally, LoS are observed to be right-skewed, so a distribution with this property should be used. In this paper, LoS is assumed to follow a Weibull distribution, which is a popular choice in survival analysis as it is robust in terms of violation of its assumptions. Therefore, the choice allows us to focus on the comparison between the different methods rather than the issues of model fit.

**Fig. 1:**
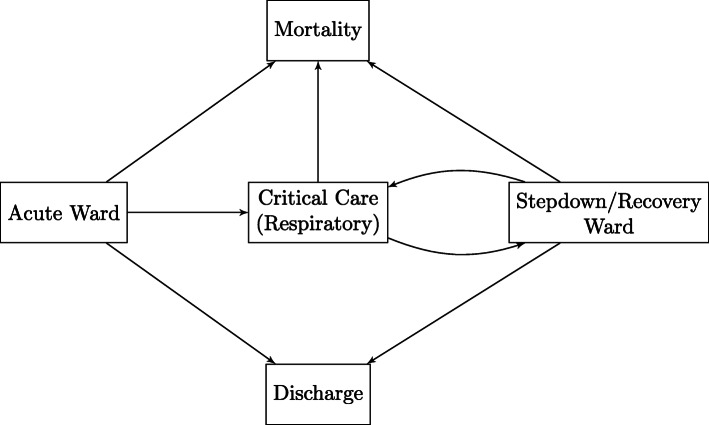
A schematic representation of the possible hospital pathways considered by our methods; at any given time, patients are considered to be in one of the five following states: Acute Ward, Critical Care, Stepdown Ward, Discharge or Mortality

Figure 1 outlines the model used to represent the hospital pathways we consider in our analysis. Allowed transitions are indicated by directed arrows between any two states. Below, we outline the survival methods we selected for our analyses. Code for all methods is available at https://github.com/thomasallanhouse/covid19-los.

### Accelerated failure time (AFT) model

In the AFT model, rather than considering all of the hospitalisation pathways shown in Fig. 1, we focus on predicting LoS in a given state, until another pre-specified event occurs. That is, we are interested in estimating the time between subsequent events in the pathway, such as from hospital admission to being admitted to ICU. We aggregate the final outcomes of death and discharge into a single outcome. This is necessary since it is not clear what the outcome will be for the censored cases in the CHESS data.

The response variable is the natural logarithm of the LoS, denoted by ln(*t*), which is explained by a vector of predictors *x*, with associated parameter vector *β*, and error term *ξ*:

1 $$\begin{array}{*{20}l} \ln(t) = x\cdot \beta + \xi. \end{array} $$

The assumed probability distribution of *ξ* defines the hazard function, i.e. the probability that a case will experience an event at time *t*, given that they have not already experienced it until time *t* [19, 20]. For *ξ* we assumed a Weibull distribution, giving the hazard function *h*(*t*)=*pλt*^*p*−1^, where *λ*= exp(−*px*·*β*) and *p* is the shape parameter defining the Weibull distribution. If *p*>1 the hazard is increasing over time, if *p*<1 the hazard is decreasing over time, and for *p*=1 the hazard is constant over time (which is equivalent to an exponential error term distribution). The predictors *x* therefore increase or decrease the hazard and so accelerate (shorten) or decelerate (lengthen) the time to event, *t*.

The AFT model explicitly takes into account cases with right-censoring [20]. Thus, the model corrects for the potential underestimation of the LoS when only a portion of patients in the sample have observed the event.

A limitation of this simple model is when there is more than one potential event of interest [18]. In this study there were two events of interest: death and discharge. These are ‘competing hazards’, i.e. if a patient experienced one they were censored for experiencing the other. We could have run the model twice, once for each event, and treated patients who experienced the other event as being censored. This would have given unbiased results if the competing hazards were independent, but, for a given patient, as the hazard of death increases, it decreases for discharge, and vice versa. For this reason we considered a model of the joint event: death *or* discharge^3^.

We fitted separate models for patients who never entered ICU versus patients who did enter ICU at some point, as these groups were expected to have different baseline hazard functions. In all models, the predictors in *x* were sex, age group and week of hospital admission (see “CHESS predictors” section).

All models were estimated using JAGS software implemented in the rjags R package [21]. For the shape parameter, we used a uniform prior, *p*∼U(0,10), which represents our lack of information on this parameter. There is not a conjugate prior simultaneously for both the shape and scale parameters in the Weibull distribution [22]. An alternative specification for this prior is a Gamma distribution [23]. However, in our tests the results were virtually the same with both priors for *p*. The scale parameter *λ* is specified via a prior for the predictors’ coefficients *β*, which is multivariate normal with mean zero and variance equal to 10, i.e. each element of *β* is distributed as $\mathcal {N}(0,10)$^4^.

### Truncation corrected method

In this method, we again focus on estimating the single LoS in a given state. We assume that LoS is given by a random variable *X*, drawn from a distribution with density function *f*_*θ*_(·), parameterised by a set of parameters *θ*. In this analysis, we assume that *X* is drawn from a Weibull distribution. We aim to determine the underlying parameters for this distribution by fitting the observed data using maximum likelihood estimation.

To use maximum likelihood estimation, we need to construct a likelihood function for the observed data. For each data point, the LoS is not directly observed. Instead, the arrival and departure dates and/or times that bracket the period of stay are observed. These correspond to two random variables, *E*_1_ and *E*_2_, linked by the LoS random variable, i.e. *E*_2_=*E*_1_+*X*. Instead of treating incomplete entries as censored, here we condition the data on observing both events. For example, if interested in the time from hospital admission to ICU admission, we condition on cases that have been admitted to hospital and to ICU. This introduces a truncation bias (See “Truncation bias” section), which needs to be corrected in the likelihood function. This approach does not take into account competing hazards, since we condition the data on observing the outcome of interest. However, this method enables LoS for different patient outcomes to be estimated, since censored cases are not included.

Our likelihood function is defined as the probability that the second event occurs on the observed date, given the time of the first event and that the second event must have occurred before the truncation date [14]. This removes censored observations since we condition on observing the second event. Therefore, we need to find

2 $$ f(E_{2}=e_{2}\mid\{E_{1}=e_{1}\}\cap\{E_{2}\leq T\})=\frac{g_{E_{1},E_{2}}(e_{1},e_{2})}{\int_{e_{1}}^{T} g_{E_{1},E_{2}}(e_{1},x)\mathrm{d}x},  $$

where $g_{E_{1},E_{2}}$ is the joint distribution of *E*_1_ and *E*_2_. The time of the second event is the time of the first event plus the delay, *E*_2_=*E*_1_+*X*. Therefore $g_{E_{1},E_{2}}=g_{E_{2}\mid E_{1}}(e_{2}\mid e_{1})g_{E_{1}}(e_{1})=f_{\theta }(e_{2}-e_{1})g_{E_{1}}(e_{1})$, which gives

3 $$ \begin{aligned} f(E_{2}=e_{2}\mid \{E_{1}=e_{1}\}\cap\{E_{2}\leq T\})&=\frac{f_{\theta}(e_{2}-e_{1})g_{E_{1}}(e_{1})}{\int_{0}^{T-e_{1}} f_{\theta}(x)g_{E_{1}}(e_{1})\mathrm{d}x}\\ &=\frac{f_{\theta}(e_{2}-e_{1})}{\int_{0}^{T-e_{1}} f_{\theta}(x) \mathrm{d}x}. \end{aligned}  $$

This can be maximised across all data points to find the maximum likelihood estimator for *θ*^5^.

This method can be used to examine LoS to individual outcomes by specifying the events, e.g. specifying that the second event is a death. Additionally, the effect of predictor variables can be analysed by sub-setting the data and then modelling the LoS of each subset.

### Multi-state model

Multi-state survival analysis extends the above two methods by permitting us to model the time to multiple outcome events in the presence of competing hazards [24, 25]. Thus, we can model complex patient pathways upon admission to hospital.

Each permitted transition in Fig. 1 is a survival model, where the instantaneous rate of transition from one state, *r*, to another state, *s*, otherwise known as the transition intensity, can be modelled similarly to hazard functions. For all transitions, we assume a Weibull AFT model, but this method can easily accommodate the use of any parametric or flexible parametric models used in standard survival analysis [19]. When there are *n*_*r*_ competing events for state *r*, a patient entering state *r* at time *t*_*j*_ has their next event at *t*_*j*+1_, which is given by the minimum of the survival times for the competing events, $\phantom {\dot {i}\!}s_{1},\ldots, s_{n_{r}}$.

The data are formatted in such a way that we have a series of event times and LoS, each corresponding to a change in state. The last of these may be observed so that the patient has entered an absorbing state, i.e. they are discharged or dead, or right-censored if the patient is still in the hospital. Therefore, the data to inform the *n*_*r*_ models consist of an indicator corresponding to whether or not the transition is observed or censored at *t*_*j*+1_. In this format, we can separate the data by transition and fit a transition-specific Weibull model to each subset^6^.

We calculate time to each transition, and the confidence and prediction intervals for these, using forward simulation together with bootstrapping [26]. Individual survival times are simulated for patients using estimates from each fitted Weibull model, and iterating through all possible transitions until all patients have reached an absorbing state or are censored at a specified maximum follow-up time. More detail on the method, including equations, is provided in Appendix D.

## Results

### Overall LoS

**Fig. 2:**
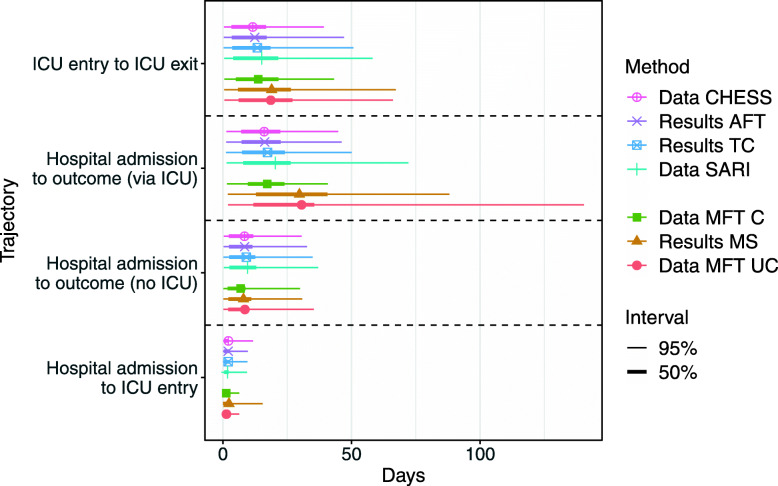
Overall Length of Stay mean estimates with 50% and 95% Predictive Intervals (PI). For CHESS and SARI data, the intervals are based on empirical percentiles. Notes: CHESS denotes data used for predictions as of 26 May 2020; SARI are the data after all patients have had seen their outcomes and missing cases have been added; MFT C denotes data with censoring; MFT UC - without censoring (after all patients have seen the outcome). Source: own elaboration using CHESS and MFT data

Table 1 and Fig. 2 show the overall estimated LoS for all three methods. Here, we present results for LoS aggregated across the outcomes of death and discharge, since this can be estimated by all three methods. In Appendix F, we consider the lengths of stay to specific outcomes. The AFT and TC estimates were all based on models adjusted for the week of admission, sex, and age group. The effect of sex was found to be small and non-significant in all of the models^7^, thus, we do not present breakdowns by it. Overall, the explanatory power of the predictor variables was only modest. They accounted for a maximum of 10 per cent of the variance in observed LoS in any of the AFT models. MS models were run without adjusting for any predictors.

**Table 1:** Overall length of stay estimates for England using the AFT and TC method, and for Manchester trusts using the MS method

Method	Hospital trajectory	Mean	SD	N
TC	Hospital admission to outcome (no ICU)	9.1	9.5	2794
TC	Hospital admission to outcome (via ICU)	17.3	13.1	2517
TC	ICU entry to ICU exit	13.4	13.8	1809
TC	Hospital admission to ICU entry	2.0	2.7	2983
AFT	Hospital admission to outcome (no ICU)	8.4	8.9	2805
AFT	Hospital admission to outcome (via ICU)	16.2	12.0	2555
AFT	ICU entry to ICU exit	12.4	12.8	1809
AFT	Hospital admission to ICU entry	2.0	2.7	2983
Multistate	Hospital admission to outcome (no ICU)	8.0	8.4	620 (786)
Multistate	Hospital admission to outcome (via ICU)	29.7	22.9	73 (101)
Multistate	ICU entry to ICU exit	18.9	18.0	92 (101)
Multistate	Hospital admission to ICU entry	2.3	4.5	101 (786)

Source: own elaboration using CHESS and MFT data. For the multi-state model, the sample size in brackets indicates the observed and censored data (including competing risks), with the first number indicating observed transitions. For TC, for sample size indicates the number of observed transitions, and for AFT the sample size is the number of observed and censored transitions

The lack of power in the predictor variables reflects the high individual-level stochasticity of LoS. Trying to predict LoS at an individual level for COVID-19 has been shown to be inaccurate [29]. The highly stochastic dynamics of infectious diseases within host, from magnitude of the initial dose to where the pathogen colonises within host, could drive differences in LoS. Therefore, the majority of variance in observed LoS are driven by the underlying stochastic process rather than explanatory variables. Although the predictors may not explain a large portion of the variance in LoS, they do have a substantial influence on the LoS distributions, with age in particular having a large influence on the expected distribution.

#### CHESS data for England

For the ICU patients (Hospital to Outcome via ICU), the shape parameters in AFT and TC methods were larger than one, implying the baseline hazard increased over time. For the non-ICU patients and LoS within the ICU, the baseline hazard remains constant in the AFT model and is slowly decreasing in TC, whereas for the Hospital to ICU admission it is decreasing in both models.

Overall, for hospital admission to final outcome, the mean LoS for patients not admitted to ICU was shorter, with an AFT mean of 8.4 (TC mean: 9.1) days, than that of patients who were admitted to ICU at some point, with an AFT mean of 16.2 (TC mean: 17.3) days. ICU admission was estimated to take 2.0 (2.0) days from hospital admission, and ICU patients were estimated to spend an average of 12.4 (13.4) days in ICU.

Standard Deviations (SD) of the estimated LoS are presented in Table 1 whereas Predictive Intervals (PIs) for the LoS in AFT and TC methods are shown in Fig. 2. The standard deviations (SD) for both the AFT and TC models are remarkably similar in depicting the large variability in the observed LoS. With the exception of the LoS from the hospital admission to outcome via ICU, all SD suggest that the waiting times till outcome are approximately exponentially distributed.

#### MFT data

Similarly to AFT and TC methods, in the MS approach, we used a Weibull distribution for each of the transition times between states in Fig. 1. Then, using fitted parameters, we used 1000 bootstraps and 10^3^ forward simulations in order to obtain estimates of the mean lengths of stay in each state, given each transition. The MFT data-based results (comparable with trajectories obtained using CHESS dataset with AFT and TC models) are presented in Fig. 2 and Table 1, along the summaries of the data.

As with the AFT and TC methods, LoS for patients admitted to ICU is longer, with a mean of 29.7 days, than that of patients not admitted to ICU, with a mean of 8.0 days. ICU admission was estimated to take 2.3 days from hospital admission and ICU patients were estimated to spend an average of 18.9 days in critical care. Taking into consideration competing hazards between stepdown and death, our mean LoS estimate for a patient in ICU is between 15.8 and 20.1 days (Table A1 in Appendix F), though in the data we observe people that have much longer critical care periods (20% of patients have over 40 days in an ICU).

### Planning with LoS

**Fig. 3:**
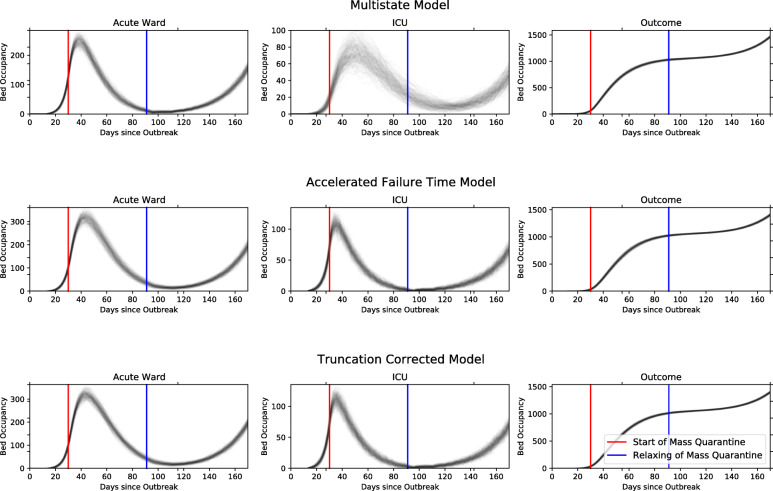
Output of our simulation for transition parameters estimated using each of our three methods, starting from 23 February, which we take to be the start of the outbreak in the UK. Source: own elaboration using CHESS and MFT data

Figure 3 predicts bed occupancy in acute ward and ICU after running our simulator with the parameter estimates of all three methods. The red and blue lines represent the implementation of, and relaxation of mass quarantine (or “lockdown”), respectively. These are considered to change the shape of the admissions trajectory to reflect that observed. We simulate hospital admissions from 23 February, first assuming exponential growth with a doubling time of 3 days, followed by exponential decay shortly after the implementation of mass quarantine. Following the blue line, we plan for a reasonable worst case scenario, and so assume a slower growth in cases with a doubling time of 15 days. Changing the assumptions used to generate hospital admissions allows us to predict and plan for any scenario of interest.

In the MS model, the hazard functions account for the competing risks of different pathways and outcomes. Therefore, hospital occupancy can be obtained by simulating the hazard functions and following the shortest transitions. In the TC and AFT models, the hazard functions are conditional on pathways and outcomes. Therefore, to simulate hospital occupancy these hazard functions need to be coupled with probabilities of each pathway. With the aggregated outcomes considered in this article, the only competing risk is whether a patient goes to ICU or not. From the MS model, the ICU admission probability is approximately 13%, so we assume the transition probability of 13% for going from the acute ward to ICU. Hospital occupancy can be then obtained by simulating the ICU probability combined with the conditional hazard functions. See Appendix E for more details.

The estimates from the AFT and TC methods yield similar predictions of bed occupancy and total observed outcomes. The MS model also gives similar predictions for acute ward and outcome but differs for ICU. The peak in bed occupancy in ICU in the MS output occurs roughly two weeks later than in the AFT and TC model outputs, and there is a slower decline after the peak. This is caused by the larger LoS estimates for the MS models as seen in Table 1 and Fig. 2.

### The effect of predictors – England

**Fig. 4:**
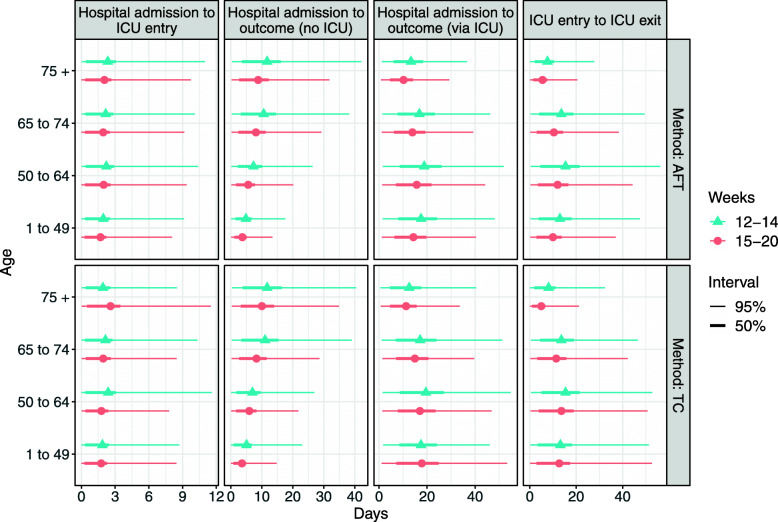
Mean Length of Stay by age and week of admission with 50% and 95% Predictive Intervals (PI). Source: own elaboration using CHESS data for England

In Fig. 4 and Table 2, we present the estimates of LoS broken down by two main predictors: age and week of admission. The mean waiting time from hospital admission to ICU entry (first column of Fig. 4) is around two days irrespective of age. For hospital admission to outcome without ICU stay (second column of Fig. 4), increasing age raises the length of stay, with length of stay around five days for the youngest age group and twelve days for the oldest, irrespective of the AFT or TC model. For individuals who go via ICU (third column of Fig. 4), the pattern with age is less clear^8^. For the first three age groups, the length of stay is roughly similar (especially AFT model), with a slight decrease in the oldest age group with respect to the first two. The 75+ age group, however, has a much shorter length of stay. A similar pattern is observed for mean LoS from ICU admission to ICU exit (fourth column of Fig. 4).

**Table 2:** Length of stay estimates with predictor variables for AFT and TC methods. Sample sizes differ due to the inclusion of censored observations in the AFT method

			AFT model			TC model		
Trajectory	Age	Weeks	Mean	SD	N	Mean	SD	N
Hospital admission to outcome (no ICU)	1 to 49	12 to 14	4.9	4.8	146	5.1	6.5	146
		15 to 20	3.7	3.6	210	3.6	4.1	210
	50 to 64	12 to 14	7.3	7.2	223	7.0	7.4	223
		15 to 20	5.6	5.4	304	5.9	5.9	304
	65 to 74	12 to 14	10.6	10.4	204	11.0	10.5	204
		15 to 20	8.1	7.9	270	8.3	7.7	266
	75 +	12 to 14	11.7	11.4	609	11.7	10.9	607
		15 to 20	8.8	8.6	839	10.0	9.4	834
Hospital admission to outcome (via ICU)	1 to 49	12 to 14	17.5	12.5	312	17.5	11.8	312
		15 to 20	14.3	10.5	267	17.8	14.0	262
	50 to 64	12 to 14	18.8	13.4	641	19.5	14.3	626
		15 to 20	15.7	11.5	467	17.0	12.1	455
	65 to 74	12 to 14	16.8	12.0	391	17.1	13.5	388
		15 to 20	13.9	10.2	225	14.9	10.2	223
	75 +	12 to 14	13.3	9.5	161	12.6	10.8	161
		15 to 20	10.2	7.6	91	11.3	8.9	90
ICU entry to ICU exit	1 to 49	12 to 14	13.0	12.8	239	13.2	14.0	239
		15 to 20	10.0	10.0	210	12.7	14.5	210
	50 to 64	12 to 14	15.4	15.2	468	15.4	14.2	468
		15 to 20	12.0	12.0	337	13.6	13.8	337
	65 to 74	12 to 14	13.6	13.4	237	13.6	12.5	237
		15 to 20	10.4	10.4	152	11.4	11.4	152
	75 +	12 to 14	7.6	7.5	109	8.1	8.9	109
		15 to 20	5.5	5.6	57	5.0	5.9	57
Hospital admission to ICU entry	1 to 49	12 to 14	2.0	2.6	340	1.9	2.5	340
		15 to 20	1.7	2.3	336	1.8	2.4	336
	50 to 64	12 to 14	2.2	2.9	732	2.4	3.3	732
		15 to 20	2.0	2.7	610	1.8	2.2	610
	65 to 74	12 to 14	2.2	2.9	421	2.1	2.9	421
		15 to 20	1.9	2.6	276	1.9	2.4	276
	75 +	12 to 14	2.4	3.1	168	1.9	2.4	168
		15 to 20	2.1	2.8	100	2.6	3.2	100

Source: own elaboration using CHESS data for England

Considering the week of admission as a predictor variable, there is less variability in LoS than in LoS disaggregated by age. For a great majority of hospital trajectories, the mean LoS seems to have decreased by, on average, 16 per cent, depending on the age group and the method used. This could be explained by potential behavioural changes in the later admission weeks. Firstly, after mass quarantine progressed individuals may have waited longer before presenting at hospital. Secondly, treatment policy has changed over the course of the outbreak, with the criteria for discharge being relaxed to ensure hospitals had capacity. Nonetheless, we also note large variability in predicted LoS both in earlier and later weeks under study.

### Model validation

Analysis of the Cox-Snell and deviance residuals for individual patients for the AFT models, in which these are well-defined [30], showed good model fit and little evidence of bias for three of the models (although there was less precision for the right-hand tails of the LoS distributions, where the effective sample size was smaller because of earlier deaths, discharges, and censoring). The exception was the model from hospital admission to ICU entry. This observed distribution was very skewed (median LoS was 0.7 days, with 20 percent over 3 days). The choice of Weibull error distribution did not represent this well, and the model showed bias in predicted LoS.

To evaluate how well the models compensated for censoring we compared the model estimated mean LoS with the data that were available during the original data collection window (i.e. including censored cases) and also the fully observed, uncensored data, which was eventually available in 2021 when all patients had left hospital. The LoS summaries based on fully observed data are presented in Fig. 2 and denoted as ‘Data SARI’ (the updated CHESS dataset), and ‘MFT UC’ (the uncensored MFT dataset). These data correct on the data used in the original analysis in three ways. Firstly, the right-censored data available at the time have been uncensored (except for a negligible proportion of patients, who we remove from the final sample). Secondly, data have been retrospectively corrected. In the original analysis, we removed the last week of data to reduce the effect of data corrections, but there could still be potential revisions. Thirdly, new patients have been added to the CHESS/SARI data. In this comparison, we only used patients admitted before 17 May 2020, to be consistent with the original data. However, in the original CHESS data, after processing this left 6208 patients, whereas in the uncensored SARI data we have 13800 patients admitted before this date. Therefore, in this validation, we investigated how the models simultaneously deal with the right-censoring, errors in the data, and case missingness of patient records.

Table A2 (see Appendix G) shows that the mean LoS from hospital admission to final outcome for patients who went into ICU at some point was on average underestimated by over five days in the original data compared to the fully observed data, and mean LoS in ICU was underestimated by over 2 days. The TC model was able to compensate for about a quarter of the underestimate in LoS for the former, and over 70 percent of the underestimate for the latter. The AFT model made smaller adjustments to the observed LoS and so captured less of the underestimate. In the original CHESS data set, we had data from 16 March 2020 to 17 May 2020, so the maximum LoS included could be 62 days. In the uncensored data, the maximum observed LoS was 245. Therefore, although the models attempted to adjust for the truncated/censored tail observations, there was insufficient data on the true extent of the tail to make the full adjustment. This illustrates how challenging it can be to estimate LoS during an emerging epidemic, even with large volumes of data.

Both TC and AFT models performed poorly for the LoS from hospital admission to ICU entry, underestimating LoS even more than the original, censored data. This is perhaps due to the Weibull distribution being inappropriate for this length of stay, and therefore struggling to capture the long tail.

The Multi-state model, on the other hand, performed well at estimating LoS for each transition (Table A2). This is in part due to local data from MFT exhibiting fewer biases than the national CHESS data so that a trust-specific LoS can be estimated with greater accuracy. The performance of the MS model can also perhaps be explained by the fact that it fully takes into account the competing risks at each transition. Of all of the LoS considered in Table 1, the maximum absolute difference between the final LoS observed in the uncensored MFT data and our estimate from the MS model is 0.98 days (from hospital admission to ICU entry), so that all of our estimates are within 1 day of the true, observed values. Again, this transition is potentially not well-captured using a Weibull hazard function.

## Discussion

### Analysis of results

#### Comparison of the three different models

In this study, we have presented three methods for estimating the LoS of patients with COVID-19 infection. Overall, the AFT and TC methods produced similar estimates for LoS for all four hospital trajectories. This is reassuring and forms an effective cross-validation of both methods and results.

The estimated mean LoS from the AFT model are shorter by around one day than the TC means, except for the Hospital-ICU entry. This might be due to the exclusion of potentially censored cases in the AFT method^9^, since it was not clear these were genuinely censored or incomplete data entries. Both methods also yielded similar predictive uncertainty about the LoS, with TC producing slightly wider predictive intervals than the AFT method. This might be explained by the explicit inclusion of the predictors in the AFT model with a joint assessment of their effect on the LoS. The TC method assumes independence between predictors and is applied to the subsets of CHESS data disaggregated by the predictor categories.

There were large differences in predicted ICU LoS between the two CHESS based methods and the MS method. The mean estimates derived using AFT and TC methods (12-13 days) were 5-6 days less than those from the MS method. The predictive intervals overlap suggesting the variability in LoS is large. However, given the focus of the paper is on comparison, and bearing in mind the MFT data is an effective census of the MFT patients and therefore that estimates are reliable in terms of the mapping of the data to the population, it is valuable to consider possible explanations for the differences in the point estimates.

These differences may reflect several substantive factors. First, MFT is one of five adult centres in the UK to have an extracorporeal membrane oxygenation (ECMO) unit. Combined with expertise in specialist respiratory care, MFT takes referrals for severe COVID-19 cases requiring ECMO treatment from other hospital trusts in the UK’s North West and Midlands regions. This higher proportion of severe cases could contribute to the longer, on average, lengths of stay observed at MFT. Unfortunately, referrals and ECMO cases cannot be separated from the MFT data, so we were unable to account for this in our analysis.

Second, the underlying data were different: the AFT and TC models used the country-wide but very incomplete CHESS data, whereas the multi-state model was based on data from just one NHS trust, but largely free of missing data. There is potentially large heterogeneity between LoS at different trusts, so data at a single trust may not reflect the national data.

Third, differences in excess bed demand from trust to trust potentially further explain discrepancies in our estimates. For trusts experiencing significant increases in demand, it is possible that they do not have the ability or resources to accurately generate daily CHESS reports which are collected in addition to routinely collected data (see Appendix A). This partially explains the case-missingness in the CHESS data.

In order to check sensitivity of the findings for the differences in the data, we evaluated the AFT model and TC method using CHESS data for Manchester University NHS Foundation Trust only. MFT contributed 53 cases with recorded LoS in ICU to CHESS. Running the AFT model on these cases gave a predicted ICU LoS of 16.5 days (SD=17.3). For the TC method, the predicted mean was 16.1 (SD= 16.7).The estimated LoS were longer than the full-sample CHESS estimates but still shorter than the predicted LoS from the MS models (18.9 days). In the MFT data, 83 cases are included. This discrepancy between the data sets could be contributing to the difference between the MS model and the AFT and TC models. Additionally, when evaluating model performance, the MS model appears to better account for the right-censoring, which could be further contributing to this discrepancy.

All methods captured the variability in the data and reflected it in the predictive distributions. This uncertainty should be taken into account when planning for the number of beds during the pandemic. For example, upper bounds of the predictive intervals can be used to construct extreme-case scenarios for the beds occupancy. These can be fed into the multi-state model to predict the number of patients in hospital at various stages of the pandemic (Fig. 3).

In the main LoS analysis above, we did not distinguish between different outcomes, such as death or discharge. Particularly in ICU, the baseline hazards for these competing hazards may be strongly diverging over time. In Appendix F, we analyse the length of stay for given outcomes using the TC and MS methods, finding that in general the length of stay to discharge is longer than to death.

#### Evaluation of model performance

When evaluating the performance of the three methods at accounting for the right-censoring, we observe different levels of performance across the methods. Using the CHESS data, the AFT model struggles to appropriately adjust for the right-censoring, resulting in an underestimate of the true distribution. The TC model does a better job at accounting for this, but still slightly underestimates the LoS. The TC model struggles to capture the true LoS because this method requires sufficient tail observations in order to adjust for the truncation bias. However, in the uncensored data there are some tail observations over twice the length of the maximum possible LoS included in the original analysis data. Therefore, the TC method does not have sufficient information to construct the true tail of the LoS distributions. The AFT model is also affected by this issue. A further complication with the AFT model is the challenge with censoring in the CHESS data. With high levels of data missingness and incompleteness at the time of the analysis, it was unclear whether cases were genuinely censored or had failed to be updated. This resulted in many censored cases being omitted from the analysis data set, leading to further underestimation of the LoS. Using the MFT data, the MS model captures the true LoS much more accurately. This model uses higher quality data, so can appropriately adjust for the censoring and the competing risks of different hospital pathways. Therefore, provided sufficiently high quality data are available, the MS approach is superior for estimating LoS during an epidemic. However, such high quality data may not be available early in a pandemic, particularly in smaller trusts. The CHESS data are not well suited for such analysis, due to the unclear case inclusion biases. This may affect the proportion of cases entering each pathway, which can interfere with the competing risks aspect of the MS model.

### Limitations of research

The CHESS dataset suffers from large amounts of case-missingness, which may lead to bias in the estimates. There appear to be three types of this. **Update delay** where a record has not been updated (with a transition) which may lead to incorrect censoring. This leads to the patient being removed from the data for some of the models. **Reporting delay** where a patient does not appear in the data at all until sometime after their admission. **Non-reporting** where no report is ever made on a patient. All three of these may cause bias in the models if they are correlated with either LoS or with extraneous variables (that are not controlled for within a given model). Another limitation of both datasets was that only cases of COVID-19 infection that led to hospital admission were included in the data. During March 2020, the hospitalised patients in England were considered to reflect the underlying population of patients with severe COVID-19 infection, but by 14 April, care-home deaths reported on death certificates caused a revision of views [31]. Those severe cases not attending hospital and COVID-19-related deaths outside of hospital may have different properties from hospitalised patients and deaths. So care should be taken in extrapolating the findings to general statements about disease progression outside of the hospital setting. Given that the goal here was to model length of stay in hospital this is less of a concern. However, change in hospitalisation practice could lead to changes in the estimates that the models produce.

Our models were also limited by the missing values in the CHESS data. A notable limitation was that around half of the cases did not have their final outcome or current status recorded. We did not know if this implied that the patient was still in hospital or whether it was an omission or whether this was a result of update delay. In either case, we had no reliable way to estimate the last time point at which the patient was observed to be in hospital, and thus these patients could not contribute to the LoS estimates. The fact that the CHESS-based LoS estimated by using the AFT models were not adjusted sufficiently to capture this suggests that many such patients were indeed still in hospital.

Compared with the AFT model, the TC method should, in theory, be less sensitive to this issue since it ignores censored cases. However, this method relies on sufficient tail observations being recorded. With the long duration of this study (over 60 days), one might expect sufficient tail observations to be included. However, with the very long lengths of stay observed in the uncensored SARI data (over 200 days), it is apparent that the original censored sample did not contain enough information on the tail of the distributions. Further complications are caused by non-random case missingness. For example, omitted cases might correspond disproportionately to tail observations, which would cause the truncation corrected method to underestimate LoS.

The strength of bias due to the truncation and censoring varies depending on the phase of the epidemic, with it having a large impact during exponential growth and lessening impact during the decay phase. The data used in this analysis is from the decay phase, so the truncation bias does not have a huge impact, and ignoring this bias would underestimate LoS by up to two days (using TC method). However, for a sample earlier in the outbreak, this underestimation may be amplified, as well as the difference in model performance. This is also true for censoring biases, since early in the outbreak the majority of cases will have censored outcomes. A large number of right-censored cases would lead to relatively large values of LoS when using the AFT model. For the purposes of this paper, we have opted not to investigate the performance of each model at different sampling dates. This is to focus on the presentation of the different methods using a single illustrative example to improve clarity. Future research could extend this in several ways, including running iteratively through the data available on different dates, modelling the impact of truncation, censoring, reporting and updating lags as the epidemic progresses.

Another issue is that clustering of patients within the NHS trusts, which were at different stages of the epidemic at different times leading to variations in pressure on capacity, could mean that there are spatial-temporal interactions in the processes driving LoS which are not captured in the models. Further, these may in turn interact with the data generating processes for CHESS with more non-reporting and reporting delays likely during high demand times. These issues could have unpredictable effects on the estimates of LoS.

With respect to the MFT data, most limitations arise due to the small absolute sample size. The multi-state method requires seeing an adequate number of patients for each state transition before any reliable modelling can take place. Indeed, although it is clinically known that stepdown to mortality is a valid transition, after applying our exclusion criteria, there were no observations of this transition occurring for patients with COVID-19 infection within this Manchester Trust. The analysis conducted in this paper therefore excluded this transition, and it is not possible to see how this influences overall hospital LoS of those patients who have an ICU episode during their hospitalisation. Together with uncommonly long ICU periods, the relative delay in the Manchester epidemic compared to other parts of the country means that MFT patients with long critical care spells are either still in ICU or only just starting to move onto stepdown. Given more weeks of data, we might be able to include stepdown to mortality in our model.

The above suggests differences between the estimates of LoS for the two datasets may therefore be due more to differences in the available data than differences in the statistical methods *per se*. It is important to acknowledge these uncertainties in the data when interpreting length of stay estimates. We further note that not only would we obtain more power in predictions through a larger amount of complete data, but also a better understanding of how the complex interactions between the virus and background risk factors affect disease severity. Additionally, inclusion criteria are slightly different between the CHESS/SARI and MFT data sets. In the CHESS/SARI data set, there is a column which indicates whether the admission was due to COVID-19. However, there is no clear definition for this, so individual hospital trusts could use different cutoff criteria, such as positive on admission or showing clear signs of COVID-19 pneumonitus. For the MFT data, we defined our own inclusion criteria, including all patients with a positive test 2 days either side of admission. At the time of the analysis (March 2020 to May 2020), there was some admissions screening at MFT, but not as widespread as the current (April 2021) requirements. Therefore, the majority of patients captured through this definition are likely to be symptomatic COVID-19 patients requiring acute care for COVID-19, rather than general admissions who return a positive swab. In both data sets we do not consider nosocomial COVID-19 cases.

## Conclusions

In this paper and its supporting materials, we provide a freely accessible set of models and tools to estimate LoS with an application to patients with COVID-19 infection. Together with a prediction of hospital admissions, which depends on the severity of outbreaks in the local area, LoS predictions can be implemented to provide organisational support within hospitals to ensure the demand for hospital and, in particular, ventilated ICU beds does not exceed availability. The models use routinely collected hospital data which are available within many national healthcare systems. Thus we anticipate our approaches will have utility across diverse healthcare systems in many different countries.

## Supplementary Information

**Additional file 1** Appendices.

## Data Availability

The data involved in this work is sensitive and therefore not publicly available. Code and parameter estimates are publicly available at https://github.com/thomasallanhouse/covid19-los.

## Abbreviations

AFT: Accelerated Failure Time
CHESS: COVID-19 Hospitalisation in England Surveillance System
ECMO: Extracorporeal Membrane Oxygenation
ICU: Intensive Care Unit
LoS: Length of Stay
MFT: Manchester University NHS Foundation Trust
MS: Multi-State
PAS: Patient Administration System
PI: Predictive Intervals
SD: Standard Deviation
SARI: Severe Acute Respiratory Infection
TC: Truncation Corrected

## Footnotes

1 Since October 2020 this has been replaced with the Severe Acute Respiratory Infection (SARI) data.

2 This can be seen in Fig. 2 by comparing the TC results to the LoS observed in the data.

3 This is not as counter-intuitive as it might sound since, although death is certainly not an equivalent outcome for the patient, our primary concern here is in length of stay regardless of outcome.

4 The model can also be estimated using maximum likelihood implemented in Stata 14 using the command streg (https://www.stata.com/manuals/ststreg.pdf)

5 We maximise this using command fminsearch in MATLAB, but it is relatively simple to implement in any language. We provide both MATLAB and Python code in the Github repository.

6 We estimate the parameter values by using maximum likelihood estimation in Python.

7 This is an interesting finding; although the severity of COVID is associated with the sex of the patient [27], the length of stay conditioning on severity is not. This has also been found with the CHESS data when controlling for other predictors, see, e.g., [28].

8 This is likely to be caused by the LoS to death (via ICU) following the opposite pattern to the LoS to discharge (via ICU). Younger age groups appear to have a longer duration until death on ICU and a shorter duration before discharge. These patterns seemingly cancel out when looking at the LoS until any outcome. This analysis is not shown here since we are focusing on length of stay in hospital rather than different outcomes.

9 For the ICU entry to ICU exit trajectory there were *n*=108 censored cases included in the model. For the trajectory from hospital admission to outcome via ICU: *n*=43, and without ICU: *n*=14. There were no censored cases in hospital admission to ICU entry.
