# Supplementary material for: Hospital length of stay for COVID-19 patients: Data-driven methods for forward planning
Source: BMC Infect Dis. 2021 Jul 22;21:700. doi: 10.1186/s12879-021-06371-6 (PMC8295642; doi:10.1186/s12879-021-06371-6)
Supplement: Supplementary file 1 — Additional file 1 Appendices. [file 12879_2021_6371_MOESM1_ESM.pdf]

Appendix A: Data generating process

The data that we analyse from MFT is routinely collected by the hospital administration teams. Details from doctors' notes and patient admissions are entered into the Trust's Patient Administration System (PAS). Patient data from PAS and WardWatcher are then aggregated together to determine an individual's entire hospitalisation pathway. We further make use of data collected by MFT on testing for COVID-19; this is, again, collected and entered manually into a database called Telepath, which is subsequently joined to the main Trust database by the Trust's data warehouse.

In addition to this routinely collected information, trusts have also been required by Public Health England (PHE) to report individual-level data on patients receiving care for acute respiratory infection and aggregate data on all COVID-19 admissions for CHESS. This information is submitted by 09:00 each day with data corresponding to the previous day. This data is compiled manually, requiring additional input from administrative staff to ensure that the data is sent on time and with the correct information. Information sent to PHE by individual trusts is then compiled into a dataset that is disseminated weekly to trusts and reported on weekly to NHS England.

Appendix B: Value-missingness in CHESS data

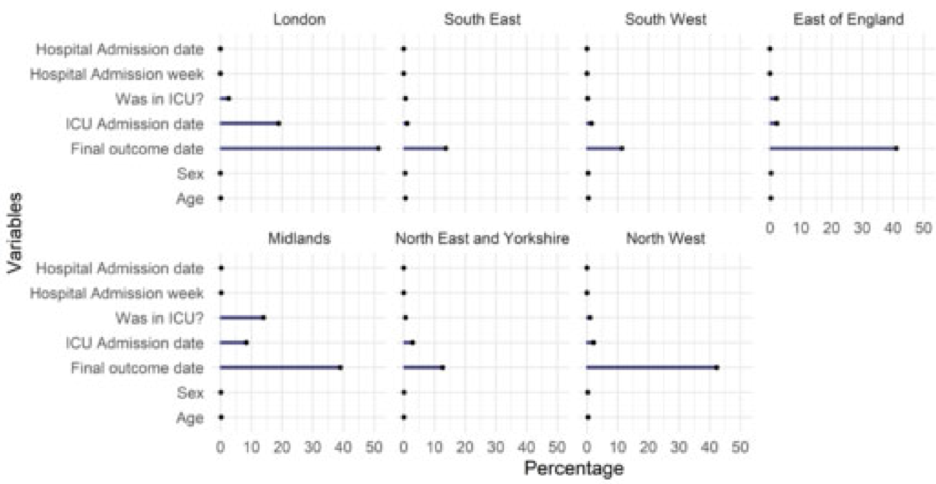

Figure A1 Percentage of values that are missing or recorded as unknown for the variables of the raw CHESS data that are used in this paper (Date and week of Hospital Admission, Was in ICU?, ICU Admission date - conditional on the patient being admitted to ICU, Final outcome date, Sex, and Age). Source: own elaboration using CHESS data for England.

Figure A1 presents the percentage of missing values in the raw CHESS data reported by NHS trusts grouped by region. London followed by Midlands have the highest percentages of missing values, while South West obtains the smallest one.

In terms of the variables, final outcome date variable has 33.6% of missing values. These will be split into cases where the missingness is because the final outcome has not yet happened and those where it has happened but has not been captured.

ICU admission date conditioned on whether the patient was admitted to ICU has 5.36% missing values. Age has 0.29% missing values, and date and week of hospital admission only have 0.06%.

By contrast, sex and the variable regarding whether a patient was in ICU or not do not have missing values. However, these do have some recorded values of "unknown" which we interpret as missing. Sex has 0.23% of unknown values, whereas the item regarding being admitted to ICU has 4.41%.

Appendix C: Data processing

We use CHESS data released on 26 May 2020 ($N = 16,138$). We first filter out 493 duplicated cases. The de-duplication rule set as follows.

**Rule**	0
**number of records**	1
**IDS are**	singular
**Date/time of admission to ICU**	Any
**Other variables**	Any values
**Action**	leave unchanged

**Rule**	1
**number of records**	2
**IDS are**	Identical
**Date/time of admission to ICU**	Identical values for dateadmittedicu
**Other variables**	different values for hospitaladmissiondate
**Action**	Include record with the earliest hospitaladmissiondate, delete the others

**Rule**	2
**number of records**	2
**IDS are**	Identical
**Date/time of admission to ICU**	Identical values for dateadmittedicu
**Other variables**	identical values for hospitaladmissiondate
**Action**	Include record with the earliest sbdate, delete the others

**Rule**	3
**number of records**	2
**IDS are**	Identical
**Date/time of admission to ICU**	Different values for dateadmittedicu
**Other variables**	ICU periods are non contiguous
**Action**	Leave all records in the file but record a different obsid for each record

**Rule**	4
**number of records**	2
**IDS are**	Identical
**Date/time of admission to ICU**	Different values for dateadmittedicu
**Other variables**	ICU periods are contiguous
**Action**	Merge the records to a single record which has the earliest hospitaladmissiondate and dateadmittedicu and the latest dateleavingicu

**Rule**	5
**number of records**	2
**IDS are**	Identical
**Date/time of admission to ICU**	One of them doesn't have dateadmittedicu
**Other variables**	
**Action**	Include record with dateadmittedicu, delete the others

**Rule**	6
**number of records**	2
**IDS are**	Identical
**Date/time of admission to ICU**	Neither has dateadmittedicu
**Other variables**	different values for hospitaladmissiondate
**Action**	Include record with the earliest hospitaladmissiondate, delete the others

**Rule**	7
**number of records**	2
**IDS are**	Identical
**Date/time of admission to ICU**	Neither has dateadmittedicu
**Other variables**	identical values for hospitaladmissiondate
**Action**	Include record with the earliest sbdate, delete the others

**Rule**	8
**number of records**	2
**IDS are**	Different
**Date/time of admission to ICU**	Identical values for dateadmittedicu, hoursadmittedicu and minutesadmittedicu (and those values are not missing).
**Other variables**	identical vales for: sex, ageyear, agemonth, hospitaladmissiondate, trustcode, postcode
**Action**	Use the record with the earliest estimateddateonset or infectionswabdate, delete the others

**Rule**	9
**number of records**	4
**IDS are**	Identical
**Date/time of admission to ICU**	identical values for dateadmittedicu for a and b, identical values for c and d and a <c
**Other variables**	a and c have identical values for hospitaladmissiondate as do b and d and a <b
**Action**	delete b and d and run the rule set over a and c

**Rule**	10
**number of records**	>1
**IDS are**	Identical
**Date/time of admission to ICU**	Any not meeting the above conditions
**Other variables**	
**Action**	mark for clerical review

The rules are applied in numerical order. In the small number of cases where rule 10 applies then this will always be where two or more of the rules need to be applied in combination.

We only analyse those patients whose records make explicit that the admission to the hospital unit was due to COVID-19. We make this assumption to exclude nosocomial cases, for whom the LoS begins when they were admitted to hospital for non-COVID-19 reasons. It does not make sense to compare these cases with LoS from COVID-19 hospitalisations. Thus, from $15,645$ deduplicated cases, $8,938$ entries were excluded.

Furthermore, we only analysed cases who were admitted to hospital from 16 March 2020 to 17 May 2020 (i.e. from week 12 to 20). Data before week 12 was omitted as this sample size was small and the treatment policy was different from that in more recent data, with patients having very long lengths of stay early in the outbreak. Data after week 20 was omitted as there are often corrections to historical data from the last week or so, so we could not treat the most recent data as reliable. This removed 317 additional cases.

Finally the omission of cases due to unknown *sex*, and negative values in *age* or recorded age of zero, and unknown (effectively missing) information regarding whether a patient was admitted to ICU or not led to a dataset of $6,208$ records. The removal of the records that have an age of 0 recorded needs further explanation. The number of records with age 0 was 725 (which compares to 15 age 1's). Many such records had characteristics that one would not attribute to newborns (e.g. obesity, smoking, diabetes, ulcers etc.). We also note that some cases with ages recorded as 0 in early versions of the CHESS dataset had been updated with non-zero ages by 26 May. It seems likely that the data entry system for CHESS has a default setting of "today" for the DOB and therefore in effect the vast majority of Age 0's were in fact cases where the age/DOB was not available when the data were entered. Hence removing these cases seems prudent.

Some LoS have zero length, where patients enter and leave ICU on the same day and only have to the day of arrival and departure recorded not the time. For such cases, we assumed the outcome occurred half a day after admission, since

instantaneous durations are unrealistic. Half a day was chosen so that these cases were not biased to either side of their recorded data. Some cases recorded hourly data for some events but not all, causing some LoS to be in $(-1, 0)$. For them, we also adjust the outcome date to half a day after admission. All patients with LoS in $(-\infty, -1]$ were discarded. In total 849 cases had their ICU admission date changed to half a day after hospital admission, 41 cases had the ICU discharge date changed to half a day after ICU admission and 199 cases had final outcome date changed to half a day after hospital admission. Therefore, the choice of how to adjust this data has the largest impact on the hospital admission to ICU length of stay, where this constitutes 28% of cases. For this length of stay, moving the adjustment to different extremes (either adding 0.1 or 0.9) changes the length of stay estimates by no more than one tenth of a day. Therefore, this data processing method does not have a substantial impact on the LoS estimates.

Appendix D: Multi-state survival analysis

Here we present the details of the multi-state survival model used in our analysis. Suppose an individual is in state u at time t, then the move that an individual makes to their next state v is governed by a set of transition intensities $q_{uv}(t) = \lim_{\delta t \to 0} \Pr(S(t + \delta t) = v \mid S(t) = u)/\delta t$. The intensity represents the the instantaneous rate of transition from state u to state v.

Data structure and transition-specific parametric models

Given the granularity of routinely collected data in hospitals, all transition times between states are observed exactly, with no additional transitions between observation times. Such data allows us to efficiently model the transition intensities parametrically, which we show here with the use of a Weibull accelerated failure time (AFT) model.

It is important to note that the data must first be structured in a specific way. In contrast to standard survival analysis, in the multi-state case, we now have a series of event times $t_1, \ldots, t_n$ for each individual, corresponding to each change in state. The last of these, may be observed so that the patient has entered an absorbing state i.e. they are discharged or dead, or right censored if the patient is still in the hospital. When there are n_u competing events for state u, a patient entering state u at time t_j, has their next event at t_{j+1} which is defined as the minimum time amongst the survival times of the competing events $v_1, \ldots, v_{n_u}$. A row is created for each transition that is possible for the patient, with an additional column consisting of an indicator corresponding to whether or not the transition is observed or censored at t_{j+1}. In this format, we can separate the data by transition and fit a transition-specific parametric model to each subset [**?**]. Our required data format is described in detail in [**?**].

Weibull AFT model

In the survival framework, for a random variable T, denoting the time until an event of interest occurs, the survival function is given by $S(t) = 1 - F(t)$, where $F(t)$ is the cumulative density function of T. The hazard function, $\lambda(t)$, is defined as the

instantaneous the instantaneous rate of occurrence of an event and is given by

$$\lambda(t) = \lim_{\delta t \to 0} \frac{P(t \le T < t + \delta t \mid T \ge t)}{\delta t}.$$

If we assume that $T \sim \text{Weibull}(k, \alpha)$, for shape parameter k and scale α, then the baseline survival and hazard functions are given by $\bar{S}(t) = \exp(-\alpha t^k)$ and $\bar{\lambda}(t) = \alpha k t^{k-1}$, respectively.

In an AFT model, predictors, $\boldsymbol{x}$, act multiplicatively on time. This in contrast to the proportional hazards model where the predictors act multiplicatively on the hazard. If we let $\phi_i = e^{\boldsymbol{\gamma} \cdot \boldsymbol{x_i}}$, where $\boldsymbol{\gamma}$ are the regression coefficients, then we get that

$$\begin{aligned}
&S_i(t) = \exp(-\alpha t_i^k \phi_i) \\
&\lambda_i(t) = \phi_i \bar{\lambda}(\phi_i t) = \phi_i k (\phi_i t)^{k-1} = \phi_i^k k t^{k-1}.
\end{aligned}$$

The model is fit using the maximum likelihood estimation (MLE) method. Formulating the likelihood for a survival model requires the consideration of both the contribution of censored and uncensored individuals. For a potentially right-censored observation, let c_i be the event indicator for the ith individual with $c_i = 1$ if an event occurred and $c_i = 0$ otherwise. Then the likelihood is given by

$$L(\gamma, k; t_i) = \prod_{i=1}^{n} \lambda_i(t_i)^{c_i} S_i(t_i).$$

Therefore it follows that the log-likelihood for such a model is given by

$$\begin{aligned}
\mathcal{L} &= \sum_{i=1}^{n} \left[c_i \log(\lambda_i(t_i)) + \log(S_i(t_i)) \right] \\
&= \sum_{i} \left[c_i (\log(k t_i^{k-1}) + k \log(\phi_i)) - (\phi_i t_i)^k \right] \\
&= \sum_{i} \left[c_i (\log(k t_i^{k-1}) + k \boldsymbol{\gamma^T x_i}) - t_i^k e^{k \boldsymbol{\gamma^T x_i}} \right].
\end{aligned}$$

Simulation/Bootstrap

In order to predict time to each transition from all states, we use a Monte Carlo simulation approach. This provides greater flexibility than computing length of stay via an integration, allowing us to predict patient pathways during an outbreak in addition to estimating length of stay in each state. As such, it creates a more powerful planning tool for hospital management. The number of simulated individuals, N, is based on COVID-19 positive hospital admissions from MFT since 23 February 2020. Individual survival times are simulated using estimates from each fitted transition-specific model, and iterating through the transition matrix until all patients have reached an absorbing state or are censored at a specified maximum follow-up time. The structure of the simulation treats the simulation as a sequence of competing hazards in the following way.

Let u be the patient's starting state, entered at time $t_u = s$ and $t_{\max}$ the maximum follow-up time of interest. For each day of interest, repeat the following to simulate paths for every new admission:

1 Let V be the set of states with an allowed transition from u and $Q_u = |V|$ be the number of possible transitions from u. While $v \in V$, let $\lambda_{uv}(t)$ be the transition intensity for the transition from u to v. Note, if $Q_u = 0$, we are in an absorbing state and stop.
2 For each possible transition, use the fitted parameter estimates of $\lambda_{uv}(t)$ to simulate a survival time, $\bar{t}_{uv}$.
3 Set $\bar{t} = \min\{\bar{t}_{u1}, \ldots, \bar{t}_{uQ_u}, t_{\max}\}$. If $\bar{t} = t_{\max}$, stop for this individual.
4 Let $u = z$ for $z \in V$ such that $\bar{t} = t_{uz}$ and set $t_u = \bar{t}$

Appendix E: Competing Hazards vs. Conditional Hazards

In this Appendix, we compare using conditional versus competing hazards within a multi-state framework. The MS model in the main text describes competing hazards, whereas the AFT and TC methods use conditional hazards. Competing hazards are perhaps more useful, but require very high quality data. If such data are not available, it may only be possible to estimate conditional hazards (where we condition on observing a given transition). However, here we demonstrate that these can be combined with estimates for the transition probabilities to obtain competing hazards. Thus, we conclude that coupling conditional hazards with transition probabilities can capture the same phenomena as a model based on competing hazards.

Consider the situation where a system has a state $X(t)$ and starts in state $X(0) = 0$ and moves to one of n states indexed by $i, j, \ldots \in [n]$, where $[n]$ is the set of integers from 1 to n inclusive.

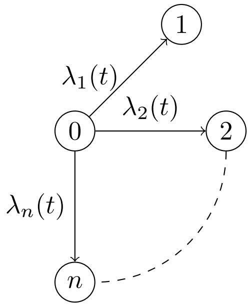

Letting $\pi_i(t) = \Pr(X(t) = i)$, we get Chapman-Kolmogorov equations

$$\frac{\mathrm{d}\pi_0}{\mathrm{d}t} = -(1 - \pi_0) \sum_{j=1}^{n} \lambda_j \ , \qquad \frac{\mathrm{d}\pi_i}{\mathrm{d}t} = (1 - \pi_0)\lambda_i \ , \qquad i \in [n] \ , \tag{1}$$

for initial conditions $\pi_0(0) = 1$, $\pi_i(0) = 1$. We now consider two models. In a **competing hazards** approach, each rate is a general integrable function, and we write these integrals as

$$\Lambda_i(t) = \int_0^t \lambda_i(u) \mathrm{d}u \ , \quad i \in [n] \ . \tag{2}$$

In a **conditional** approach, we add an additional random variable, I, which is the state that the system will move to, i.e. $\lim_{t\to\infty} X(t)$. We then let

$$\lambda_i(t) = \mathbf{1}_{\{I=i\}} r_i(t) \ , \tag{3}$$

where $r_i(t)$ is the rate of going from 0 to i conditional on that being the event that happens. Integrals of these rates are defined as

$$R_i(t) = \int_0^t r_i(u) \mathrm{d}u \ , \quad i \in [n] \ . \tag{4}$$

Our aim is to show that the two approaches (competing and conditional) can be calibrated to give consistent results for quantities of interest.

One result needed for consistency is on the final outcome probabilities:

$$\Pr(I = i) = \lim_{t\to\infty} \Pr(X(t) = i) =: \pi_i^\infty \ , \quad i \in \{0\} \cup [n] \ , \tag{5}$$

where we have allowed for the possibility that the system may never leave the state 0, although for most of the parametric models we consider that will not be the case. Imposing consistency of the probability of being in state 0 over time, by integrating (1) and using the law of total probability for the conditional model,

$$\pi_0(t) = \exp\left(-\textstyle\sum_{i\in[n]} \Lambda_i(t)\right) = \pi_0^\infty + \textstyle\sum_{i\in[n]} \pi_i^\infty \exp\left(-R_i(t)\right) \ . \tag{6}$$

Substituting (6) back into (1), we can then obtain (also using the law of total probability)

$$\frac{\mathrm{d}\pi_i}{\mathrm{d}t} = \lambda_i(t) \left(1 - \exp\left(-\textstyle\sum_{i\in[n]} \Lambda_i(t)\right)\right) = \pi_i^\infty r_i(t) \left(1 - \exp\left(-R_i(t)\right)\right) \ , \qquad i \in [n] \ . \tag{7}$$

We can then solve both (6) and (7) simultaneously using the Ansatz

$$\lambda_i = \mathcal{C} \pi_i^\infty r_i(t) \ . \tag{8}$$

This ensures that (7) is satisfied, then substituting into (6) we obtain

$$\exp\left(-\mathcal{C} \textstyle\sum_{i\in[n]} \pi_i^\infty R_i(t)\right) = \pi_0^\infty + \textstyle\sum_{i\in[n]} \pi_i^\infty \exp\left(-R_i(t)\right) \ , \tag{9}$$

and hence

$$\mathcal{C} = \frac{\log\left(\pi_0^\infty + \sum_{i\in[n]} \pi_i^\infty \exp\left(-R_i(t)\right)\right)}{-\sum_{i\in[n]} \pi_i^\infty R_i(t)} \ . \tag{10}$$

This demonstrates that it is possible to capture the same phenomena of interest in the two models given appropriate calibration.

Appendix F: Additional results

In the main results section, we provided estimates for the LoS until any outcome. This was chosen to facilitate comparison between the different methods. In addition to this LoS, the TC method and the MS method can be used to estimate the length of stay until given outcomes such as discharge or death.[1] In this section, we compare estimates for these LoS. Again, pathways are disaggregated by whether the individual went via ICU. Here we choose to omit further predictor variables such as age or week of admission from the TC method to aid comparison.

Table A1 shows the comparison. For the LoS without ICU, the two methods give similar estimates. Using the TC method on the CHESS data predicts that LoS to mortality without ICU is slightly shorter than that to discharge, whereas the MS model on the MFT data predicts vice versa. This might be explained by the small sample size for the MFT data and the different demographic profile of the wider population captured by CHESS. The two methods predict very different LoS on ICU, with ICU to mortality being more than 5 days longer in MS (15.8 days) to that predicted by TC (10.2 days). Similarly, the LoS from ICU to stepdown, where individuals are discharged from ICU back to the general ward, is also longer with the MS model. As with the main results, this could most likely be explained by the presence of a much higher proportion of ECMO patients in the MFT data than in the CHESS data. The LoS from stepdown to discharge is similar for the two methods, with 7.9 from TC and 6.2 from MS. We are unable to estimate stepdown to ICU from CHESS due to the small number of such cases present in the data.

Table A1 Length of stay estimates to given outcome for England using the truncation corrected (TC) method, and for the Manchester Trust using the multi-state (MS) method. Source: own elaboration using CHESS and MFT data.

Method	Hospital trajectory	Mean	95% Confidence Interval
TC	Acute Ward to ICU	2.0	(1.9, 2.1)
TC	Acute Ward to Discharge	9.4	(8.9, 9.9)
TC	Acute Ward to Mortality	8.3	(7.8, 8.9)
TC	ICU to Stepdown	16.6	(15.6, 17.6)
TC	ICU to Mortality	10.2	(9.7, 10.7)
TC	Stepdown to ICU	NA	NA
TC	Stepdown to Discharge	7.9	(7.5, 8.3)
Multistate	Acute Ward to ICU	2.2	(1.9, 2.9)
Multistate	Acute Ward to Discharge	7.8	(7.0, 8.6)
Multistate	Acute Ward to Mortality	8.7	(7.5, 9.8)
Multistate	ICU to Stepdown	20.1	(15.9, 25.1)
Multistate	ICU to Mortality	15.8	(12.0, 21.5)
Multistate	Stepdown to ICU	2.2	(1.1, 7.6)
Multistate	Stepdown to Discharge	9.9	(7.2, 14.0)

Figure A2 shows the output of our simulator on bootstrapped MFT data using the complete multi-state model in Figure 1. The red line represents true data that is plotted against 200 bootstrap simulations using fitted estimates for the transitions.

[1]A univariate AFT model is not fit for estimating LoS for competing hazards, such as for death and discharge.

Day 0 is taken to be 23 February, which we have taken to be the start of the national outbreak for the UK.

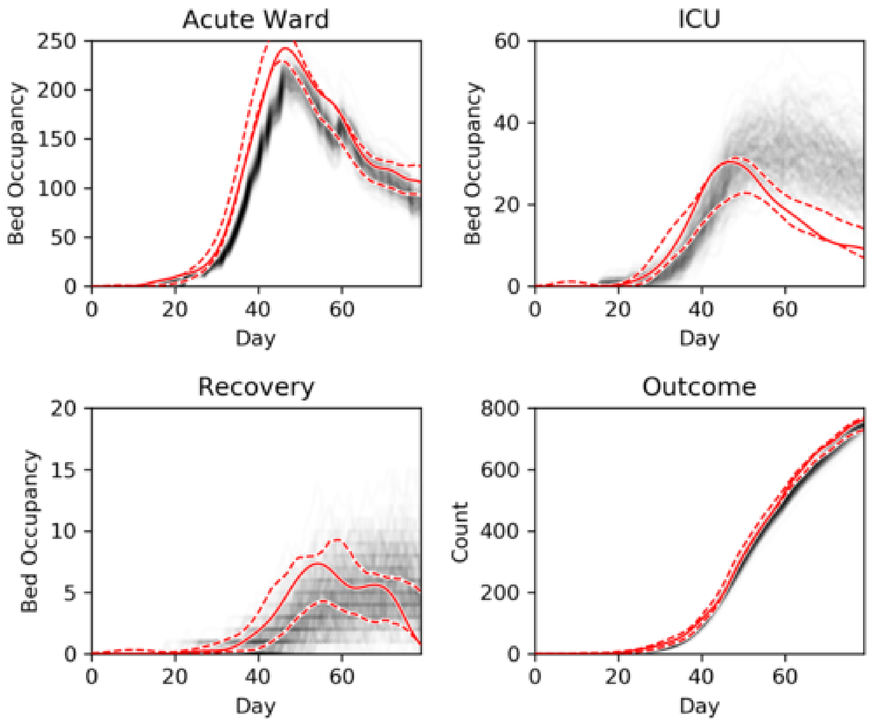

Figure A2 Output of our simulation using the MS method (black cloud of points) on MFT data (red lines). Source: own elaboration using MFT data.

Appendix G: Model Validation Results

Table A2 compares our mean LoS estimates from each model with the observed LoS in the data. For both the CHESS and MFT datasets we show the LoS from the censored data that was available prior to the 17th of May, which was the cutoff date for our original analysis, and from the uncensored data obtained once all patient outcomes had been observed. We also show the difference between the two, thereby showing by how much taking the observed mean LoS from the data underestimates the true LoS. For each model we then record the estimated LoS for each pathway and calculate both the absolute error when compared to the true uncensored LoS from the data as well as the error as a percentage of the LoS underestimate.

Table A2 Validation of our models against the fully observed, uncensored (UC) data. For the accelerated failure time (AFT) and truncation corrected (TC) models we record both the absolute difference between our model mean estimates and the uncensored data from CHESS and as a percentage of the difference between the censored (C) and uncensored data. For the Multistate (MS) model we perform the same analysis, but against the data from MFT.

		Admission to ICU entry	ICU entry to ICU exit	Admission to Outcome (via ICU)	Admission to Outcome (no ICU)
	LoS (C)	2.12	11.58	16.03	8.37
CHESS Data	LoS (UC)	2.18	14.16	21.19	9.41
	LoS Underestimate	0.06	2.58	5.16	1.04
	Mean	2.00	12.40	16.20	8.40
AFT model	Difference	0.18	1.76	4.99	1.01
	% of adjustment	-200.00	31.78	3.29	2.88
	Mean	2.00	13.40	17.30	9.10
TC model	Difference	0.18	0.76	3.89	0.31
	% of adjustment	-200.00	70.54	24.61	70.19
	LoS (C)	1.30	13.72	17.21	6.86
MFT Data	LoS (UC)	1.35	18.58	30.57	8.52
	LoS Underestimate	0.05	4.86	13.36	1.66
	Mean	2.33	18.93	29.73	7.97
MS model	Difference	-0.98	-0.35	0.84	0.55
	% of adjustment	2060.00	107.20	93.71	66.87
